# An mHealth Intervention to Reduce Gestational Obesity (mami-educ): Protocol for a Randomized Controlled Trial

**DOI:** 10.2196/44456

**Published:** 2023-02-15

**Authors:** Delia Indira Chiarello, Fabian Pardo, Jessica Moya, Maricela Pino, Andrea Rodríguez, María Eugenia Araneda, Ayleen Bertini, Jaime Gutiérrez

**Affiliations:** 1 Cellular Signaling and Differentiation Laboratory, School of Medical Technology Faculty of Medicine and Science Universidad San Sebastián Santiago Chile; 2 Metabolic Diseases Research Laboratory, Interdisciplinary Center for Research in Territorial Health of the Aconcagua Valley Center for Biomedical Research Universidad de Valparaíso San Felipe Chile; 3 School of Medicine, Campus San Felipe Faculty of Medicine Universidad de Valparaíso San Felipe Chile; 4 School of Nutrition and Dietetics Faculty of Sciences for Health Care Universidad San Sebastián Santiago Chile; 5 School of Obstetrics Faculty of Sciences for Health Care Universidad San Sebastián Santiago Chile; 6 School of Psychology Universidad Católica Silva Henríquez Santiago Chile; 7 PhD Program Doctorado en Ciencias e Ingeniería para La Salud Faculty of Medicine Universidad de Valparaíso Valparaíso Chile

**Keywords:** gestational obesity, mHealth, primary care, randomized controlled trial

## Abstract

**Background:**

The World Federation of Obesity warns that the main health problem of the next decade will be childhood obesity. It is known that factors such as gestational obesity produce profound effects on fetal programming and are strong predictors of overweight and obesity in children. Therefore, establishing healthy eating behaviors during pregnancy is the key to the primary prevention of the intergenerational transmission of obesity. Mobile health (mHealth) programs are potentially more effective than face-to-face interventions, especially during a public health emergency such as the COVID-19 outbreak.

**Objective:**

This study aims to evaluate the effectiveness of an mHealth intervention to reduce excessive weight gain in pregnant women who attend family health care centers.

**Methods:**

The design of the intervention corresponds to a classic randomized clinical trial. The participants are pregnant women in the first trimester of pregnancy who live in urban and semiurban areas. Before starting the intervention, a survey will be applied to identify the barriers and facilitators perceived by pregnant women to adopt healthy eating behaviors. The dietary intake will be estimated in the same way. The intervention will last for 12 weeks and consists of sending messages through a multimedia messaging service with food education, addressing the 3 domains of learning (cognitive, affective, and psychomotor). Descriptive statistics will be used to analyze the demographic, socioeconomic, and obstetric characteristics of the respondents. The analysis strategy follows the intention-to-treat principle. Logistic regression analysis will be used to compare the intervention with routine care on maternal pregnancy outcome and perinatal outcome.

**Results:**

The recruitment of study participants began in May 2022 and will end in May 2023. Results include the effectiveness of the intervention in reducing the incidence of excessive gestational weight gain. We also will examine the maternal-fetal outcome as well as the barriers and facilitators that influence the weight gain of pregnant women.

**Conclusions:**

Data from this effectiveness trial will determine whether mami-educ successfully reduces rates of excessive weight gain during pregnancy. If successful, the findings of this study will generate knowledge to design and implement personalized prevention strategies for gestational obesity that can be included in routine primary care.

**Trial Registration:**

ClinicalTrials.gov NCT05114174; https://clinicaltrials.gov/ct2/show/NCT05114174

**International Registered Report Identifier (IRRID):**

DERR1-10.2196/44456

## Introduction

The World Obesity Federation warns that the main health problem of the next decade will be childhood obesity [1,2]. Obesity and its consequences have their origin in intrauterine life [3]. A study showed that the consumption of “junk” food (high in energy and poor in micronutrients) during pregnancy was a predictor of high birth weight and the child’s future preference for foods high in fat, sugar, and salt [4]. The risk of obesity in childhood is increased if the mother has a high level of adiposity or develops gestational diabetes, or if the baby is not breastfed or experiences poor nutrition that leads to delayed birth [2]. Excessive gestational weight gain (eGWG) is a predictor of overweight and obesity in children in the short-, medium-, and long-term, with evidence of effects up to 21 years after delivery [5-7]. In this sense, according to Barker theory, nutrition and other environmental factors to which the mother is exposed during pregnancy alter intrauterine life [8]. Gestational obesity produces deep effects on the programming of the fetal genome, thereby inducing changes in the prenatal metabolism that extend to the postnatal period, which is also associated with increased susceptibility to developing cardiovascular and metabolic diseases in adulthood [5-7,9,10].

eGWG during early pregnancy has been repeatedly associated with increased adiposity in childhood and adolescence [11-15]. Thus, adequate maternal nutrition during pregnancy is the window of opportunity to strengthen the health status of the mother and fetus and reduce the risk of adverse birth outcomes, such as preterm birth and low birth weight associated with eGWG [16]. The first trimester of pregnancy is critical for short-term interventions to break the “transmission” of obesity from one generation to the next [17]. For many women, pregnancy is a powerful motivator for self-care, as they are more aware of personal risks and have a strong emotional response toward adopting risk-reducing behaviors [18]. Quantitative literature suggests that the key to maintaining a healthy pregnancy and the primary prevention of maternal-fetal complications is establishing healthy dietary behaviors during gestation [19,20]. Even though many women are aware of the importance of healthy eating during pregnancy, the lack of nutritional self-care knowledge represents a substantial barrier to weight control during this stage [21,22]. Nutrition education improves nutritional knowledge, thus influencing practices aimed at achieving adequate nutrition [22]. Several studies recommend the continuous use of educational interventions on nutrition during pregnancy in routine obstetric practice to improve the quality of life of pregnant women and their offspring [6,23-25]. Regarding this, a study performed on the Chilean population evaluated the efficacy of a low-intensity, high-coverage nutritional intervention at the primary health care level [26]. This high-coverage intervention included training health professionals on nutritional recommendations, counseling pregnant women on diet and physical activity, and appropriate referral to primary health care centers’ dieticians. The study found that the intervention reduced gestational weight gain and has the potential for successful scale-up [26].

New strategies to engage the target population are required to maintain nutritional recommendations as a priority in the daily decision-making (top of mind) of pregnant women [27]. Interventions based on behavioral theories provide a greater understanding of the underlying mechanisms that determine health-related behavior change and have the potential to be more effective in promoting adherence to weight gain control [28]. The social cognitive theory [29,30] considers the social dimension of the individual since each individual has a belief system and the capacity for self-motivation and positive behavioral changes [29,31,32]. Therefore, technologies such as mobile phones can be useful to effectively address this problem. Mobile phone ownership is widespread among all sectors of society, regardless of socioeconomic status, which gives us the possibility to introduce the mobile health (mHealth) intervention as an important component of future health care practice [32].

**Figure 1:**
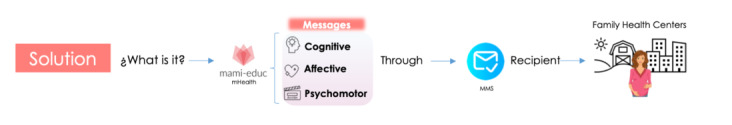
Schematic diagram of the proposed solution. The solution called "mami-educ" consists of sending cognitive, affective, and psychomotor messages through MMS to pregnant women that assist family health centers in urban and rural areas. MMS: multimedia messaging system.

mHealth programs are potentially more effective than face-to-face interventions since they make it possible to cover a larger part of the population; moreover, face-to-face interventions are more labor-intensive, time-consuming, and expensive. Furthermore, they are especially useful during public health emergencies, like the recent COVID-19 pandemic [33]. According to the World Health Organization, mHealth has been defined as “medical and public health practice supported by mobile devices, such as mobile phones, patient monitoring devices, personal digital assistants, and other wireless devices” [34]. Mobile phones are personal, portable, and connected devices [30]. This means that messages can be proactively delivered directly to people, while face-to-face activities or visiting a web page require “going” to look for the information, that is, the self-determined motivation of pregnant women. In this sense, a recent meta-analysis that included 12 randomized controlled trials (RCTs) conducted in an adult population of different ages and conditions demonstrates the efficacy of interventions based on mobile phone apps for weight loss [35]. The combined findings of 10 of the studies included in the meta-analysis showed a substantial reduction in BMI (−0.45 kg/m^2^) and body weight (−1.07 kg) in the intervention group [35]. A recently published RCT that aimed to evaluate the efficacy of a digital health intervention on maternal and perinatal outcomes in pregnant women with obesity who attended the maternal-fetal department of the Hospital-Clinic of Barcelona [31] demonstrates that the digital intervention, which was delivered by the midwife through an app, including advice on gestational weight gain and physical activity, found that gestational weight gain was lower and physical activity increased in obese pregnant women in the intervention group [31]. Hence, we aim to evaluate the effectiveness of an mHealth intervention, based exclusively on mobile phone–delivered messages directed to the cognitive, emotional, and psychomotor behavior, called mami-educ, to reduce excessive weight gain in pregnant women who attend family health care centers in urban and predominantly rural areas (Figure 1). The intervention will last 12 weeks and consists of sending messages through the multimedia messaging system (MMS), with food education addressing the cognitive, affective, and psychomotor domains. According to the literature, the basic factors for adopting a healthy behavior are the perceived susceptibility to the perceived threat of a disease, the perceived consequence, such as severity, the perception of the positive benefits of the action, the perceived barriers to action, and the confidence in the ability to succeed, which is perceived as self-efficacy for action [36]. Thus, the messages that address the cognitive domain of learning are aimed at helping pregnant women understand the benefits of a healthy diet and the harm of a diet rich in fats and sugars during pregnancy, while the affective domain will address the beliefs, barriers, and opportunities to achieve the consumption of healthier foods and contribute to the health of the mother-child binomial. Behavior change is initiated and sustained when people feel that they can execute the desired behavior (ie, self-efficacy) and have a reasonable expectation that the behavior will lead to the desired outcome [37]. In this sense, the psychomotor domain seeks to promote self-efficacy by delivering messages about preparations with adequate nutrition for pregnancy and planning actions to avoid eGWG.

## Methods

### Study Design and Participants

The design of the intervention corresponds to a classic RCT, a design that allows us to prospectively study pregnant women, in which it is intended to compare the effect of the intervention versus the control condition, to establish a cause-and-effect relationship. This design allows us to reduce the risk of comparability bias. The study will be carried out with pregnant women who control their pregnancy in Family Health Centers (CESFAM) of the Metropolitan Region (the capital city of Chile) and the Aconcagua Valley, Valparaiso Region (88 km to the north of the capital). The CESFAM work under the Comprehensive Health Model with a Family and Community Approach, focusing on prevention and health promotion, and are responsible for basic health care, including home care and health rehabilitation actions. These centers focus on families and communities, give importance to community participation, and work with a primary health team that attends to the whole family in health and sickness throughout the life cycle.

### Control Group

Pregnant women enrolled in the prenatal control program receive routine care at the CESFAM.

### Intervention Group

Pregnant women enrolled in the prenatal control program receive routine care at the CESFAM and messages on nutrition (intervention).

### Inclusion Criteria

Chilean- or Spanish-speaking immigrant pregnant women older than 18 years who receive care at the 6 CESFAM in El Bosque, Metropolitan Region and in Segismundo Iturra CESFAM in San Felipe, Aconcagua Valley, Valparaiso Region; who have gestational ages ≤12 weeks (first trimester); who have a single pregnancy; who declare to have a normal pregnancy at the time of starting the intervention; who agree to be randomized; and who have voluntarily signed the informed consent can participate in this study. The pregnant women who participate in the study must have a mobile device that allows the use of MMS.

### Exclusion Criteria

Exclusion criteria include multiple pregnancies, conditions requiring a special diet, participants with psychiatric illnesses or other prepregnancy pathology, and a history of recurrent miscarriages.

### Sample Size Calculation

The total sample consists of 511 participants, which will be divided into 2 groups. In the El Bosque CESFAM, 280 pregnant women were recruited, with an estimated dropout rate of 30% (based on the pilot study carried out) to guarantee the required sample size of 210 pregnant women with a confidence level of 90% and a 5% error. At Segismundo Iturra CESFAM, 231 pregnant women were recruited, with the same estimated dropout rate, to ensure a sample size of 178 pregnant women with a confidence level of 90% and a 5% error. For the calculation of the sample size, the official records of the DEIS regarding the number of pregnant women between 20 and 44 years of age who were treated for prenatal control in the CESFAM of the El Bosque commune and Segismundo Iturra were considered. The recruitment period will be 6 months.

### Randomization and Masking

The pregnant women who meet the inclusion criteria are invited to participate in the study, as are those who accept and sign the informed consent form. These pregnant women receive the link to the diagnostic survey on their telephones and are then randomized to be included in the intervention. At the end of each month of the recruitment period, the researchers will randomize the pregnant women who were recruited during that month. A stratified randomization will be carried out to ensure a sample that is as balanced as possible. The sample will be divided into 4 strata according to their pregestational weight, classifying them as underweight (BMI<18.5 kg/m^2^), normal weight (BMI 18.5-24.9 kg/m^2^), overweight (25-29.9 kg/m^2^), and obese (BMI≥30 kg/m^2^). Simple random selection will be carried out then by generating a table of random numbers without repetition, from 1 to n, and deciding a priori that it will be read from left to right, with even numbers assigned to the control group and odd numbers to the intervention group.

A single-blind study will be carried out since the CESFAM personnel who will oversee the recruitment will be blinded to the randomization of the subjects to avoid bias in routine care.

### Procedures

The recruitment of pregnant women will be done for a period of 6 months. The study will end when all the participants with intervention have completed the 12-week message period.

Immediately after randomization, the researchers will send the MMS with the link that leads to a web-based survey. The survey will be the same for the control group and intervention group and will recollect demographic information, nutritional habits, and physical activity. Pregnant women in the intervention group who respond to the survey will receive the intervention messages by MMS.

Routine care visits will be scheduled usually by CESFAM’s midwifery professionals. Such visits should occur monthly until the 28th week of gestation, biweekly during the 28-36 weeks of gestation, and weekly until delivery. During prenatal control, the nutritional evaluation is carried out according to the technical standard, and a referral to a nutritionist is made if necessary. These care visits may also include participation in the “Elige Vivir Sano” and “Chile Crece Contigo” programs and everything associated with the prenatal control program.

### Intervention

The intervention consists of 3 MMS messages with nutritional information per week for 12 consecutive weeks. The messages were reviewed by a perinatal psychologist. The messages will be sent as follows: Monday at 3 PM, the message that addresses the cognitive domain will be sent; Tuesday at 3 PM, the message that addresses the affective domain will be sent; and Thursday at 3 PM, messages that address the psychomotor domain, together with the weekly user satisfaction survey, will be sent. This delivery scheme was validated in the pilot study that was carried out. The 12 topics related to nutrition during pregnancy will be addressed, 1 will be addressed per week, and 3 messages will be sent for each topic. At the beginning of each month, messages will be sent to pregnant women who were recruited during the immediately previous month. In the development of the project and to prevent pregnant women from incurring additional expenses for participating in the intervention, we will directly recharge the phones of the users who participate in the study.

### Follow-up

The team of midwives will keep a record of the weight of the pregnant women who attend their prenatal checkups monthly. Therefore, the monitoring of the weight of both groups will be done through the midwives in order not to request sensitive data via MMS.

### Dropouts

A record of the number of pregnant women who drop out of the study will be kept, as well as their characteristics for subsequent statistical analysis.

### Primary and Secondary Outcomes

The primary outcome will be to assess the effectiveness of the mHealth mami-educ intervention in reducing the incidence of eGWG compared to routine care. Secondary outcomes will include identifying the barriers and facilitators influencing weight gain in pregnant women to compare the maternal outcome of pregnancy, in terms of complications developed during pregnancy (pre-eclampsia, gestational diabetes, and gestational hypertension), and the perinatal outcomes (prematurity and birth weight) of the pregnant women in the control and intervention groups.

### Statistical Analysis

The data analysis plan will be governed by the intention-to-treat principle, as we will include all participants who were randomly assigned after recruitment, assuming that participants who drop out are treatment failures. The effectiveness of the treatment will be measured using the chi-square test (χ^2^), comparing the intervention group with the control group in terms of the percentage of women who are classified as having excessive weight gain at the end of pregnancy. To confirm the effectiveness of the intervention, a significant decrease (*P*<.001) of women with eGWG in the intervened group will be expected. For data analysis, the statistical software GraphPad Instat 3.0b and GraphPad Prism 6.0f (GraphPad Software Inc) will be used. *P*<.001 (determined by a 2-sided test) will be considered statistically significant.

### Ethics Approval

This research will be conducted in accordance with the Declaration of Helsinki. The study was approved by the Scientific Ethics Committee of the Aconcagua Health Service (protocol number: 26/2021, approved October 12, 2021) and the Scientific Ethics Committees of the South Metropolitan Health Service (protocol number: 07-27012022, approved February 7, 2022). Informed consent will be obtained from all participants invited to participate in the study before proceeding with the initial survey. The data will be treated respecting the ethical principles that guarantee the anonymity of the participants.

## Results

The study was funded by the National Research and Development Agency of Chile in December 2021. The data collection began on May 2, 2022. Study staff have been engaged in activities associated with study including enrollment and data collection. As of November 30, 2022, a total of 222 pregnant women have been admitted for the first prenatal appointment, 127 meet the inclusion criteria, and 76 agree to participate and answered the diagnostic survey. The expected timeframe for completing recruitment and data analysis is November 2023.

## Discussion

### Principal Findings

It is well known that pregestational obesity has been recognized as a risk factor for several perinatal complications, such as fetal macrosomia, gestational diabetes, hypertensive disorders, failed induction, premature rupture of membranes, and cesarean section [38]. Nevertheless, this risk is increased when eGWG is present [39,40]. Prevention strategies in primary care are key to fighting this problem; in this regard, strategies that target behavior change by promoting self-efficacy have been proposed [37]. A recent study conducted focus groups with 66 women between 16 and 24 weeks’ gestation [19], and the participants expressed that the difficulties in operationalizing the information in the guidelines and that provided by health care providers were one of the important barriers to self-care. For this reason, care providers’ advice on nutrition and physical activity was perceived as minimal and ineffective. We propose the integration of social cognitive theory with mHealth to provide nutritional information to pregnant women in primary care. In this study, the structure of the messages addresses 3 learning domains (cognitive, affective, and psychomotor) that promote self-efficacy [37]. The hypothesis of this study is that the mHealth intervention called mami-educ is effective in reducing eGWG in pregnant women who attend family health care centers in an urban area and another predominant rural area. From a public policy perspective, effective health communication is very important; mHealth provides fast, easy-to-use, and cost-effective communication. We designed this study to address the gap in evidence-based primary prevention strategies regarding the use of mHealth to reduce eGWG. Overall, this proposal has the potential to reduce the incidence of perinatal complications through adequate gestational weight gain. To ensure the reproducibility of the study for future research and reduce the risk of comparability bias, the design of the intervention corresponds to a classic RCT. This design allows us to carry out a prospective study of pregnant women in which it is intended to compare the effect of the intervention versus the control condition to establish a cause-and-effect relationship.

The results of this study may serve to implement evidence-based primary prevention strategies and help a broader group of pregnant women maintain adequate weight gain during pregnancy and prevent pregnancy pathologies associated with obesity. This would ultimately influence health care costs. In Chile, it has been described that the cost of overweight and obesity increases to US $455 million by year, representing 2% of the total health budget, which means that obesity costs 4-fold higher than normal weight health care [41]. Through this observation, the projected health costs for 2030 related to overweight and obesity will reach 3.4% of the total health budget (US $3 billion). Furthermore, it is estimated that a weight reduction of 7%-10% in the obese population would translate into a 40% decrease in spending on obesity-associated comorbidities [42]. Thus, promoting adequate weight gain during pregnancy in women with obesity could improve maternal, fetal, newborn, and offspring health and could significantly reduce public health care costs. Lastly, the data collected in this study can play a central role in the development of public policies based on mHealth directed exclusively to the population of pregnant women.

### Limitations

A limitation of this study is the reliance on smartphones to access interventions. In addition, the recruitment of pregnant women must occur during the first trimester of gestation, and this may be hindered because some pregnant women begin prenatal care in the second trimester. Another limitation is that the pregnant woman may decide not to interact with the messages by blocking the number of the intervention.

## Appendix

Multimedia Appendix 1 Evaluation report.

## Abbreviations

CESFAM: Centro de Salud Familiar
eGWG: excessive gestational weight gain
mHealth: mobile health
MMS: multimedia messaging system
RCT: randomized controlled trial

## Data Availability

The data sets generated and analyzed during this study will be available from the corresponding author on reasonable request following the completion of the trial and publication of the main outcomes paper.
