# Supplementary material for: An mHealth Intervention to Reduce Gestational Obesity (mami-educ): Protocol for a Randomized Controlled Trial
Source: JMIR Res Protoc. 2023 Feb 15;12:e44456. doi: 10.2196/44456 (PMC9978990; doi:10.2196/44456)
Supplement: Multimedia Appendix 1 [file resprot_v12i1e44456_app1.pdf]

Lunes, 12 de Julio de 2021

INFORME DE EVALUACIÓN

XVIII Concurso Nacional de Proyectos de Investigación y Desarrollo en Salud, Fonis 2021 - FONDO DE FOMENTO AL DESARROLLO CIENTIFICO Y TECNOLOGICO

IDENTIFICACIÓN DE LA POSTULACIÓN

**Folio**	SA21I0099
**Título**	Evaluación e implementación de una intervención mHealth para combatir la obesidad gestacional en mujeres de la Región Metropolitana y el Valle de Aconcagua.
**Postulante**	Delia Chiarello Peñaranda
**Instituciones**	**Beneficiaria Principal** UNIVERSIDAD SAN SEBASTIAN<br>**Otra Beneficiaria** UNIVERSIDAD DE VALPARAISO<br>**Entidad asociada** ILUSTRE MUNICIPALIDAD EL BOSQUE

PUNTAJE DE EVALUACIÓN

Sección	Ponderación	Nota Promedio	Promedio Sección
**1. I.- Relevancia del Tema y Caracterización del Problema**	20%	5.00	1.00
**2. II.- Solución e Investigación.**	20%	4.87	0.97
**3. III.- Metodología, Ética y Planificación.**	35%	4.75	1.66
**4. IV.- Resultados, Implementación y Difusión.**	10%	4.10	0.41
**5. V.- Capacidad de Gestión y Asociatividad.**	15%	4.50	0.67

Nota Final: 4.71

PARÁMETROS PARA PROCESO DE CALIFICACIÓN

Los Proyectos serán evaluados con notas de 0 a 5, siendo 0 el mínimo y 5 el máximo.
Cada Capítulo será analizado en base a los criterios proporcionales en las bases, referidos a la información que se detalla en el formulario de postulación correspondiente.
Los Capìtulos de evaluación son:

Sección	Ponderación
**1. I.- Relevancia del Tema y Caracterización del Problema**	20%
**2. II.- Solución e Investigación.**	20%
**3. III.- Metodología, Ética y Planificación.**	35%

Sección	Ponderación
**4. IV.- Resultados, Implementación y Difusión.**	10%
**5. V.- Capacidad de Gestión y Asociatividad.**	15%

Cada Capítulo será evaluado y calificada usando la siguiente escala:

Calificación	Concepto	Nota Promedio
**0**	**No Califica**	La propuesta no cumple/aborda el criterio bajo análisis o no puede ser evaluada debido a la falta de antecedentes, o información incompleta.
**1**	**Deficiente**	La propuesta no cumple/aborda adecuadamente los aspectos del criterio o no hay graves deficiencias inherentes.
**2**	**Regular**	La propuesta cumple/aborda en términos generales los aspectos del criterio, pero existen importantes deficiencias.
**3**	**Bueno**	La propuesta cumple/aborda correctamente los aspectos del criterio, aunque requiere ciertas mejoras.
**4**	**Muy Bueno**	La propuesta cumple/aborda los aspectos del criterio de muy buena manera, aun cuando son posibles ciertas mejoras.
**5**	**Excelente**	La propuesta cumple/aborda de manera sobresaliente todos los aspectos relevantes del criterio en cuestión. Cualquier debilidad es muy menor.

Nota: Es posible usar medios puntos, pero no fracciones menores

DETALLE DE LA EVALUACIÓN

1. I.- Relevancia del Tema y Caracterización del Problema (20%)

1.1 1.1 RELEVANCIA DEL TEMA

**Descripción Pregunta**	a) ¿Se justifica adecuadamente la pertinencia y relevancia de abordar este problema de salud en el momento actual? ¿Es la temática relevante para el desarrollo de la Salud Pública nacional?<br>b) Evalúe la vigencia de la temática. ¿Se justifica adecuadamente? ¿La realización del proyecto será un aporte para la salud de la población y/o para el sistema de salud del país o localidad?<br>c) ¿Se explica por qué el proyecto debe ser abordado mediante un proyecto de investigación en salud?
**Porcentaje Pregunta**	50%

Nota Pregunta:

5.00

Justificación:

Si se justifica adecuadamente la pertinencia y relevancia de abordar este problema de salud en el momento actual y la temática es altamente relevante para el desarrollo de la Salud Pública nacional, es vigente y se justifica adecuadamente

y la realización del proyecto será un aporte para la salud de la población en estudio y para el sistema de salud del país. Se explica claramente por que el proyecto debe ser abordado mediante un proyecto de investigación en salud.

1.2 1.2 PLANTEAMIENTO DEL PROBLEMA

**Descripción Pregunta**	Se debe evaluar la concordancia con los lineamientos generales del concurso, así como exponer las bases conceptuales que dan sustento a la sección metodológica y la forma de abordar el problema:<br>a) ¿Se establece de forma adecuada la conceptualización necesaria para el desarrollo del proyecto?<br>b) ¿Se establece, de manera clara, cuál es el problema de salud abordado? ¿cuáles son las condiciones que determinan su existencia? y, ¿cuál es la relevancia de abordarlo en el momento presente?<br>c) ¿Se plantea un vacío de conocimiento que justifique la realización del proyecto?<br>d) ¿Se cuantifica el problema? De no ser así, ¿se justifica por qué no es posible su cuantificación?<br>e) ¿Se establece la concordancia con los lineamientos generales establecidos en las bases del concurso?<br>f) ¿Se entregan antecedentes que definan por qué la estrategia para abordar el problema, en este momento y condiciones, es la más adecuada?
**Porcentaje Pregunta**	20%

Nota Pregunta:
5.00

Justificación:
La conceptualización del tema que se quiere abordar con el proyecto de investigación es apropiado. Se establece, de manera clara, cuál es el problema de salud abordado, logrando visualizar sus determinantes. El problema de salud abordado es claro "obesidad en mujeres gestantes", se presenta evidencia que lo respalda, tanto a nivel nacional como internacional, que permiten establecer claridad en la temática a abordar por esta investigación, permite sustentar su desarrollo y aplicación. no existe un vacío de conocimiento en la materia, de hecho existe bastante evidencia a nivel internacional de los distintos tratamiento que se pueden aplicar, lo importante es poder aplicar las experiencias extranjeras aterrizaras a nuestro contexto nacional y a nuestra cultura, con el fin de contribuir de forma positiva a los objetivos estratégicos en salud que como país nos hemos planteado. El problema de salud se encuentra perfectamente cuantificado y se ha presentado dicha información en forma adecuada.

1.3 1.3 ESTADO DEL ARTE

**Descripción Pregunta**	Respecto de la revisión de la literatura:<br>a) ¿Es pertinente? ¿Es actualizada? ¿Aborda los avances en la resolución del problema a nivel nacional e internacional?<br>b) Dados los antecedentes planteados por los autores ¿Queda claro

	cuánto se sabe del problema de salud que se quiere abordar?<br>c) En su opinión ¿Los antecedentes planteados por los autores abarcan todas las formas en que se ha abordado el problema en el país y en el mundo? ¿Se mencionan proyectos en desarrollo en la misma línea de investigación y las propias?
**Porcentaje Pregunta**	30%

Nota Pregunta:
5.00

Justificación:
El estado de arte y la literatura es pertinente y actualizada. También aborda los avances en la resolución del problema a nivel nacional e internacional. Queda suficientemente claro cuánto se sabe del problema de salud que se quiere abordar. Los antecedentes planteados por los autores abarcan las formas en que se ha abordado el problema en el país y en el mundo. Mencionan su propia investigación de un piloto previo, en la misma línea de investigación.

2. II.- Solución e Investigación. (20%)

2.1 2.1 SOLUCION PROPUESTA Y ESCENARIOS DE APLICABILIDAD

**Descripción Pregunta**	Se debe evaluar la originalidad, nivel de resolución del problema abordado y nivel de aplicabilidad<br>a) La descripción de la solución para abordar la propuesta: ¿Es adecuada? ¿Es completa? ¿Se justifica por qué abordar el problema de esta forma?<br>b) ¿La solución propuesta aporta al logro de la solución final del problema?<br>c) ¿La realización del proyecto será un aporte para la salud de la población chilena y/o al sistema de salud del país o para alguna localidad o población específica?<br>d) ¿Se analizan y comparan en forma crítica y realista las situaciones que se registrarán con y sin el desarrollo de la investigación propuesta?
**Porcentaje Pregunta**	50%

Nota Pregunta:
5.00

Justificación:
La propuesta es original e innovadora. La descripción de la solución para abordar la propuesta es adecuada y completa, justifica por qué abordar el problema de esta forma. La solución propuesta aporta al logro de la solución final del problema, sería un aporte para la salud de este grupo y podría ser extrapolable a la población chilena pudiendo aportar a disminuir la obesidad en la gestación, la innovación de utilizar dispositivos móviles masificados es un estrategia que aportaría a la solución parcial del problema. Se analizan y comparan las situaciones que se registrarán con y sin el desarrollo de la investigación propuesta.

2.2 2.2 PREGUNTA DE INVESTIGACIÓN E HIPÓTESIS O SUPUESTO DE INVESTIGACIÓN

**Descripción Pregunta**	a) En relación a la pregunta de investigación: ¿está fundamentada en la sección del marco conceptual? ¿está formulada de manera clara y precisa?<br>b) La hipótesis o supuestos de investigación ¿se formulan claramente? ¿orientan de forma adecuada al propósito de la investigación? ¿se señala de forma explícita y clara el enfoque u orientación de la investigación?
**Porcentaje Pregunta**	25%

Nota Pregunta:

5.00

Justificación:

La pregunta de investigación presentada esta bien fundamentada tanto en la relevancia del tema y su caracterización como así también en el planteamiento del problema, su formulación es clara y muy precisa. La hipótesis se encuentra bien formulada y en coherencia con la pregunta de investigación, además permite conocer de forma adecuada la finalidad y orientación de la investigación a desarrollar.

2.3 2.3 OBJETIVO GENERAL Y OBJETIVOS ESPECÍFICOS

**Descripción Pregunta**	a) ¿Están formulados de manera coherente entre sí? ¿Son claros? ¿Son completos y concisos?<br>b) ¿Son cumplibles y realizables en los tiempos y condiciones de la propuesta de investigación planteada?
**Porcentaje Pregunta**	25%

Nota Pregunta:

4.50

Justificación:

El objetivo general esta bien planteado, los objetivos específicos son claros y coherente entre sí, sin embargo, el objetivo especifico N°3 podría considerarse mas una actividad que un objetivo , además debería incluirse las 3 dimensiones evaluadas en este punto (cognitivo, afectivo y psico-motriz)
Son cumplibles y realizables en los tiempos y condiciones de la propuesta de investigación planteada.

3. III.- Metodología, Ética y Planificación. (35%)

3.1 3.1 METODOLOGIA Y PROCEDIMIENTOS

**Descripción Pregunta**	Se debe evaluar la pertinencia del diseño, su alcance y los

	procedimientos que lo operacionalizan. Respecto del diseño:<br>a) ¿Se menciona y describe de manera explícita el diseño de la investigación propuesta?<br>b) ¿Se justifica por qué es el diseño más adecuado para responder a la pregunta de investigación, así como su alcance y la temporalidad del estudio?<br>c) Según su opinión, ¿Es el diseño propuesto el más adecuado para responder a la pregunta de investigación y corroborar hipótesis y/o supuestos?<br>RESPECTO DE LA METODOLOGIA Y PROCEDIMIENTOS:<br>a) ¿Se incluyen y describen adecuadamente todos los procedimientos que permitirán operacionalizar el diseño propuesto? ¿Se señalan de forma clara los aspectos relevantes/críticos de la metodología para cumplir con el diseño propuesto y para responder la pregunta de investigación planteada (outcome primario o principal)?<br>b) ¿Se describe la población de donde provendrán los participantes del estudio? ¿Los criterios de selección de la muestra? ¿El cálculo y el tamaño de la muestra? ¿Se explicitan los criterios de inclusión y exclusión?<br>c) ¿Se plantean los mecanismos o la información necesaria para asegurar reclutamiento de los participantes?<br>d) ¿Se describe la adecuación de las técnicas y métodos contemplados en el plan de análisis respecto de los objetivos del estudio?<br>e) ¿Se describen las técnicas de producción de información y el plan de análisis de datos de forma detallada y secuencial?<br>f) ¿Se señalan los aspectos relevantes/críticos para evaluar la viabilidad de la propuesta y sus resultados adicionales?<br>g) En su opinión ¿es una propuesta viable?
**Porcentaje Pregunta**	75%

Nota Pregunta:

5.00

Justificación:

El diseño es pertinente y realizable, se propone un diseño de intervención de tipo ensayo clínico aleatorizado clásico que es el adecuado, ya que permitirá comparar el efecto y el valor de esta intervención en salud. El diseño de la intervención propuesta y a través de un ECA permitirá dar respuesta a la pregunta de investigación planteada, incluye su alcance y temporalidad propuesta. El diseño propuesto ECA clásico es el más riguroso y válido que permitirá evaluar la efectividad de la intervención mHealth propuesto en este proyecto, dando respuesta a la hipótesis planteada por los investigadores, Están incorporados en la propuesta y descritos en forma adecuada, todos los procedimientos necesarios para llevar a cabo esta investigación, Se señalan claramente los Outcome Primario y secundarios, que son coherentes con la pregunta de investigación y con los Objetivos generales y específicos, se describe claramente la población de donde se obtendrá la muestra que permitirá el desarrollo de la investigación, el piloto realizado les permitió conocer que un 30% aproximadamente puede desertar, están claramente señalados y explicados los criterios de inclusión y exclusión, siendo estos los apropiados dada la naturaleza y orientación del proyecto, se describe el cálculo y el tamaño de la muestra y es el apropiado que permite asegurar el desarrollo de la propuesta. Como es una población que esta bajo control, asociados a un CESFAM, esto permitirá asegurar el reclutamiento de las participantes, por otro lado y gracias al pilotaje, se conoce cual es el la deserción probable y sobre esa basa trabajar con la muestra

necesaria, los objetivos propuestos tanto el general como los específicos, que están claramente definidos, permitiendo que los métodos contemplados para el análisis de los resultados sean coherentes, posibilitarán el adecuado desarrollo de esta investigación. El plan de análisis estadístico propuesto es el adecuado, se aprecia una sistematización de los procesos, se considera pruebas para dar la validez interna como la externa necesaria para el desarrollo de la investigación. Se señalan los aspectos críticos para la viabilidad de la propuesta. Es una propuesta absolutamente viable.

3.2 3.2 ANÁLISIS DE LAS IMPLICANCIAS ÉTICAS

**Descripción Pregunta**	En este capítulo, los autores, deben realizar un autoanálisis de las implicancias éticas que supone la ejecución del proyecto, es decir, la estrategia de preservación de los principios éticos y bioéticos que se pueden ver comprometidos durante la ejecución del proyecto, y las acciones que desarrollará el proyecto para mitigar dichos riesgos.<br>CONSIDERACIONES ETICAS:<br>a. El equipo de investigación debe analizar las implicancias éticas de estudio, de manera coherente y consistente con el protocolo de estudio, y con sus características y contextos, y no de manera general.<br>b. Los investigadores deben señalar los principales dilemas éticos, indicando cómo los abordarán y cómo reducirán los posibles efectos no deseados.<br>c. Se deberá explicitar si el estudio requerirá consentimiento informado y/o asentimiento (no es necesario adjuntarlos en esta etapa)<br>d. Señalar caminos de acción frente a temas emergentes que afecten algún principio ético (por ej: detección de una enfermedad o situación de salud de riesgo)<br>En este sentido y en su opinión:<br>a) El autoanálisis realizado por los autores en las 4 secciones del capítulo (análisis de riesgo-beneficio; resguardo de la confidencialidad; consentimiento/asentimiento informado; autorizaciones institucionales requeridas) ¿es adecuado?<br>b) ¿Se indican las autorizaciones institucionales que serán requeridas para la ejecución del proyecto? en su opinión ¿son las necesarias?<br>Para la evaluación de este capítulo se recomienda revisar los aspectos más importantes relativos a la investigación con seres humanos en el siguiente link: https://www.conicyt.cl/fondecyt/2012/10/31/bioetica/
**Porcentaje Pregunta**	15%

Nota Pregunta:
4.00

Justificación:

Se presenta un análisis bien detallado de las implicancias éticas del proyecto presentado. El autoanálisis presentado por los proponentes respecto de análisis de riesgo-beneficio, resguardo de la confidencialidad, consentimiento informado y autorizaciones institucionales requeridas, es adecuado y pertinente a lo
requerido para este tipo de estudio. se recomendaría inscribirlo como ensayo clínico. Se presentan la mayoría de las autorizaciones, sin embargo, no se presenta la carta de autorización de Centro de salud Familiar Segismundo Iturra del Valle de Aconcagua, es importante presentarla ya que es necesaria.

3.3 3.3 PLAN DE TRABAJO

**Descripción Pregunta**	a) ¿Se consideran todas las etapas y/o actividades necesarias para el desarrollo del proyecto? Las etapas o actividades ¿concuerdan con las señaladas en la metodología y procedimientos? ¿Se definen en forma detallada?<br>b) ¿Se definen plazos viables para la ejecución general del proyecto?<br>c) ¿se consideran tiempos administrativos en la ejecución del proyecto? ¿se consideran los plazos para la tramitación de la documentación necesaria para la ejecución del proyecto?
**Porcentaje Pregunta**	10%

Nota Pregunta:
4.00

Justificación:
El plan de trabajo presenta todas las etapas y actividades necesarias para el proyecto coherentemente con la metodología y procedimientos. Se recomienda agregar el tiempo para la publicación comprometida.

4. IV.- Resultados, Implementación y Difusión. (10%)

4.1 4.1 IMPLEMENTACIÓN DEL (DE LOS) PRODUCTO(S) ESPERADO(S)

**Descripción Pregunta**	Se debe evaluar la calidad de los resultados y productos esperados, la factibilidad de que sean logrados dado los antecedentes entregados en los capítulos anteriores y la coherencia con la solución propuesta.<br>Respecto de la implementación:<br>a) ¿Se definen claramente los destinatarios finales del (los) producto (s) esperado (s)? y ¿Dónde serán implementados?<br>b) ¿Se describe, de manera adecuada, la estrategia de implementación del (los) resultado (s) o producto (s) esperado (s) y sus mecanismos de transferencia?<br>Nota: Toda producción científica, tales como publicaciones, libro, presentación en congreso, debe ser considerada en este apartado.
**Porcentaje Pregunta**	80%

Nota Pregunta:
4.00

Justificación:
Los destinatarios finales están claramente identificados y explicitados. También los productos esperados , así como serán implementados en forma detallada. igualmente, la estrategia de implementación y sus mecanismos de trasferencia y estrategia , haciéndose notar que gran parte de la implementación se ira realizando durante el proyecto, aunque la implementación post proyecto estará definida de acuerdo a los resultados obtenidos. Consideran producción científica , prestaciones a congresos y talleres a personal , se recomienda que se amplíe el marco para la difusión a tomadores de decisión y pacientes.

4.2 4.2 ACTIVIDADES DE DIFUSIÓN

**Descripción Pregunta**	Las actividades de difusión deben estar claramente identificadas. Se debe evaluar la factibilidad de su realización en el plazo de ejecución del proyecto, así como su pertinencia.
**Porcentaje Pregunta**	20%

Nota Pregunta:
4.50

Justificación:
Se describen claramente las actividades de difusión propuestas, siendo apropiadas dada la contingencia nacional producto de la pandemia, se mencionan talleres asociados, congresos, presentación a revistas para posible publicación y un simposio. Todas las actividades son pertinentes, viables, acorde a los plazos de ejecución del proyecto. Se sugiere reforzar actividades de difusión no académicas.

5. V.- Capacidad de Gestión y Asociatividad. (15%)

5.1 5.1 CAPACIDAD DE GESTIÓN Y ANTECEDENTES CURRICULARES

**Descripción Pregunta**	a) Respecto de la conformación del equipo de investigación ¿Es esta la adecuada para dar cumplimiento a la propuesta? ¿Cuenta el equipo con la formación y experiencia necesaria?<br>b) Se debe evaluar la pertinencia de las actividades y funciones asignadas a cada integrante del equipo de investigación, personal técnico, de apoyo y personal administrativo ¿Las responsabilidades individuales se encuentran correctamente asignadas?
**Porcentaje Pregunta**	40%

Nota Pregunta:
5.00

Justificación:

El equipo de investigación posee las competencias necesarias, que aseguran la ejecución de cada una de las actividades del proyecto de investigación, cuentan con la experiencia necesaria, además ligados con la línea investigativa asociada al proyecto. Las actividades funciones presentadas para cada uno integrante del equipo de investigadores, junto a la dedicación horaria propuesta, son las adecuadas y se encuentran bien asignadas

5.2 5.2 PARTICIPACIÓN DE INVESTIGADORES EN FORMACIÓN

**Descripción Pregunta**	Investigador en formación: persona que está en etapa inicial de sus habilidades de desarrollar proyectos de investigación autónomamente, podría ser un estudiante de pregrado, postgrado o un académico.<br><br>¿Se exponen estrategias adecuadas de formación de investigadores pertinentes al estudio?<br>¿Se explicitan las actividades de formación, roles, y quien supervisa al investigador en formación?<br>Nota: 1) Se excluyen labores que se pueden contratar, como encuestadores, etc.
**Porcentaje Pregunta**	20%

Nota Pregunta:

5.00

Justificación:

Se describe la incorporación de investigadores en formación de pregrado y postgrado, se explicitan las actividades de formación, roles, y quien supervisa a los investigadores en formación.

5.3 5.3 PROPUESTA ECONÓMICA

**Descripción Pregunta**	a) ¿La propuesta económica considera todos los costos relevantes para la realización del proyecto? ¿Los costos considerados son pertinentes?<br>b) En caso de que corresponda ¿se consideran gastos de traslado de los pacientes? ¿Se presenta una distribución adecuada de los recursos?<br>Nota: Para evaluar esta pregunta, debe revisar la PLANILLA DE COSTOS en los documentos adjuntos.
**Porcentaje Pregunta**	20%

Nota Pregunta:

3.50

Justificación:
La propuesta económica es pertinente y considera los costos relevantes para la realización del proyecto, sin embargo, la Institución beneficiaria solo consideran gasto en personal y no el gasto no incremental, No se describe el traslado de pacientes el aporte de locomoción no aparecen en la planilla

5.4 5.4 ASOCIATIVIDAD

**Descripción Pregunta**	a) ¿La propuesta considera la participación de Instituciones Asociadas?<br>b) En caso que no lo considera ¿debería considerarlas?<br>c) En caso que sí las considere, ¿Estas son pertinentes? ¿Su participación aporta a asegurar la obtención de los resultados? ¿Estas comprometen algún tipo de aporte a la propuesta?
**Porcentaje Pregunta**	20%

Nota Pregunta:
4.00

Justificación:
El proyecto de investigación cuenta con una adecuada asociatividad, cuenta con las instituciones beneficiarias y con instituciones asociadas los servicios de salud pertinentes, asegurando el logro de los objetivos y resultados. Falta la carta de compromiso del Centro de salud familiar Segismundo Iturra Del Valle de Aconcagua

5.5 CONCLUSION FINAL

**Descripción Pregunta**	Ingrese comentarios generales de la evaluación. Además, indique si recomienda la adjudicación de la propuesta o no. Indique si se requiere realizar exigencias o recomendaciones al proyecto (indique cuáles).
**Porcentaje Pregunta**	0%

Respuesta:
Sí

Justificación:
Se sugiere adjudicar con las siguiente CONDICION DE ADJUDICACIÓN:
1.- Deben inscribirlo como ensayo clínico, y presentar la carta de autorización del Centro de salud Familiar Segismundo Iturra del Valle de Aconcagua.
